# Effect of *Saccharomyces boulardii* on Liver Diseases: A Systematic Review

**DOI:** 10.3390/microorganisms12081678

**Published:** 2024-08-15

**Authors:** Roman Maslennikov, Nona Benuni, Anna Levshina, Farida Adzhieva, Tatyana Demina, Alina Kucher, Ekaterina Pervushova, Evgeniya Yuryeva, Elena Poluektova, Oxana Zolnikova, Evgenii Kozlov, Alexey Sigidaev, Vladimir Ivashkin

**Affiliations:** 1 Department of Internal Medicine, Gastroenterology and Hepatology, Sechenov University, Moscow 119435, Russiaalinakucher1608@gmail.com (A.K.);; 2 Scientific Community for the Promotion of the Clinical Study of the Human Microbiome, Moscow 119435, Russia; 3 Laboratory of Immunopathology, Department of Clinical Immunology and Allergy, Sechenov University, Moscow 119435, Russia; kozlov-evgeny@bk.ru; 4 Department of Clinical Disciplines, Tyumen State Medical University, Tyumen 625023, Russia

**Keywords:** dysbiosis, probiotics, intestinal permeability, leaky gut, gut–liver axis, microbiome, microbiota

## Abstract

We aimed to systematize the results of published studies on the use of *Saccharomyces boulardii* (SB) for the treatment of various liver disorders (CRD42022378050). Searches were conducted using PubMed and Scopus on 1 August 2022. The PubMed search was updated on 15 June 2024. The review included sixteen studies: ten experimental animal studies (EASs) and six randomized controlled trials (RCTs). The CNCM I-745 strain was used in 68.8% of the included studies. SB reduced the severity of many manifestations of cirrhosis, and lowered the Child–Pugh scores in RCT. SB reduced the serum concentrations of TNF-α, IL-1β, IL-6, and IL-4 in animals with metabolic dysfunction-associated steatotic liver disease (MASLD); lowered the serum TNF-α and IL-6 levels in experimental cirrhosis in rats; and reduced the CRP levels in decompensated cirrhosis. The EAS of MASLD revealed that SB reduced liver steatosis and inflammation and lowered the liver expression of genes of TNF-α, IL-1β, interferon-γ, and IL-10. In studies on experimental cirrhosis and MASLD, SB reduced the liver expression of genes of TGF-β, α-SMA, and collagen as well as liver fibrosis. SB reduced the abundance of Escherichia (Proteobacteria), increased the abundance of Bacteroidetes in the gut microbiota, prevented an increase in intestinal barrier permeability, and reduced bacterial translocation and endotoxemia.

## 1. Introduction

Chronic liver diseases are an important cause of disability and increased mortality [1,2]. The main chronic liver diseases are alcoholic and metabolic dysfunction-associated steatotic liver diseases (MASLD) as well as hepatitis B and C [1,2]. Liver damage in these diseases is manifested by an increase in the serum activity of liver enzymes ALT and AST as well as the development of cholestasis, the biomarkers of which are the serum levels of GGT and alkaline phosphatase (ALP). In addition, in MASLD, an important role is played by the disturbance of carbohydrate and lipid metabolism, which is manifested by hyperglycemia, hypercholesterolemia, hypertriglyceridemia, hyperinsulinemia, an increase in total body weight as well as the mass of visceral and somatic fat. In alcoholic liver disease and MASLD, there is also an increase in the size and mass of the liver due to an increase in the fat content in it (liver steatosis). Inflammation, oxidative stress, and fibrosis develop in the liver tissue in chronic liver diseases that naturally lead to the development of their final stage, namely cirrhosis. Cirrhosis is characterized by decreased liver function (manifested by hypoalbuminemia, hyperbilirubinemia, hypocoagulation, and hepatic encephalopathy) and portal hypertension (manifested by ascites, hypersplenism, esophageal varices, and worsening hepatic encephalopathy).

It is now established that the state of the intestine and its microbiota exerts a great influence on liver function whether disease is present or not. The gut microbiota form a mass of biologically active substances; these include lipopolysaccharides (LPS, endotoxin), short-chain fatty acids (SCFAs), secondary bile acids, tryptophan metabolites, ammonia, and hydrogen sulfide, amongst others. By acting on specific receptors and directly interfering with metabolic pathways, they modulate metabolism as well as the immune, nervous, and endocrine systems, resulting in a pluripotent effect on the entire body. The liver is the first organ to encounter blood that is rich in these intestine-derived metabolites, and is one of the main points of application of their action. This concept is called the gut–liver axis [3,4,5,6,7]. As an example, in cirrhosis, the abundance of harmful Proteobacteria in the gut microbiota increases, the level of beneficial bacteria of the Clostridia class decreases, and the intestinal barrier is weakened, which leads to bacterial translocation and endotoxinemia, stimulating systemic inflammation and hyperdynamic circulation. The latter aggravates the course of portal hypertension. Many of these changes in the gut–liver axis, although less pronounced, are observed in chronic liver diseases at the pre-cirrhotic stage [3,4,5,6,7].

In recent years, the gut–liver axis has become the target of a number of drugs; these include probiotics (i.e., live bacteria used for therapeutic purposes). Our recent review revealed that probiotics may produce beneficial effects in a variety of liver diseases [8]. However, different probiotics have different effects. In this regard, these drugs should be considered not only as an entire group; rather, each probiotic microorganism should be considered separately. Among these, *Saccharomyces boulardii* (SB), a probiotic yeast that has proven its effectiveness in the treatment of *Clostridioides difficile* [9,10,11,12] and *Helicobacter pylori* [13,14,15] infections, is particularly attractive to researchers. Recently published studies have demonstrated the positive roles played by these probiotics in the treatment of cirrhosis [16,17]. The aim of the present review was to systematize the published results of studies on the use of *Saccharomyces boulardii* for the treatment of various liver disorders.

## 2. Materials and Methods

The protocol for this systematic review was registered with PROSPERO (CRD42022378050). The systematic review was performed in accordance with PRISMA guidelines.

The search was conducted using PubMed and Scopus on 1 August 2022. The search expression was “*Saccharomyces boulardii*” for PubMed and “TITLE-ABS-KEY (saccharomyces AND boulardii) AND (LIMIT-TO (DOCTYPE, “ar”) OR LIMIT-TO (DOCTYPE, “le”))” for Scopus. A total of 923 publications were found in PubMed, and 1395 publications were found in Scopus. After excluding 692 duplicates, 1623 unique publications were analyzed. After the initial analysis was completed, the PubMed search was updated on 15 June 2024, and 172 publications from 2022–2024 were added to the analysis, bringing the total number of unique publications to 1795. Unfortunately, the authors did not have access to Scopus on this latter date, so the list of publications in this database was not updated.

This list further excluded publications that were not original articles (reviews, editorials, clinical cases), those that did not contain data on the effect of SB on liver disorders, and non-controlled studies. Studies in which SB was administered together with other probiotics but no groups were administered SB as the only probiotic were also excluded. Studies that included birds or other non-mammals were also excluded as the physiologies and pathologies of such animals can differ greatly from those of humans.

We tried to review the full text of articles wherever possible.

The selection of publications and data extraction were carried out independently by Roman Maslennikov and a group of co-researchers (Nona Benuni, Anna Levshina, Farida Adzhieva, Tatyana Demina, Alina Kucher, Ekaterina Pervushova, and Evgenia Yuryeva). Roman Maslennikov looked through all the found publications; each researcher from that group was a co-researcher for a part of the publication list. All disagreements between them were resolved by consensus. If consensus could not be reached, the referee was the main project manager, Vladimir Ivashkin.

## 3. Results

**Figure 1:**
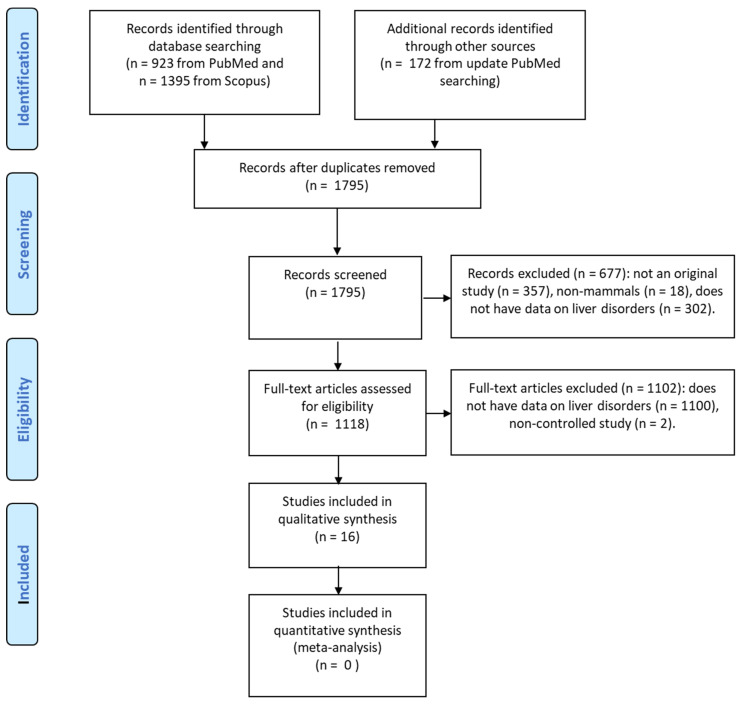
PRISMA flow diagram.

After excluding all irrelevant publications, the systematic review included sixteen studies: ten experimental animal studies (EASs) and six randomized controlled trials (RCTs) in humans (Figure 1, Table 1, Table 2 and Table 3) [16,17,18,19,20,21,22,23,24,25,26,27,28,29,30,31]. There were single EASs on giardiasis, Salmonella enteritis infection, obstructive jaundice, and D-galactosamine-induced liver injury. Two EASs were conducted on cirrhosis, and four were conducted on MASLD. Of the six RCTs, two examined SB in cirrhosis, and four examined SB in neonatal hyperbilirubinemia (Table 3).

The CNCM I-745 strain, which is the most widely used in clinical practice, was used in 11 (68.8%) included studies. A different strain was used in one study. The strains used were not described in the remaining four studies (Table 1 and Table 3).

Published data on the effects of SB on the serum levels of ALT, AST, GGT, ALP, bilirubin, albumin, glucose, insulin, cholesterol, triglycerides, biomarkers of systemic inflammation, oxidative stress, and bacterial translocation in various liver disorders were systematized (Table 2 and Table 3). Published data on the effect of SB on the severity of oxidative stress, cell damage, inflammatory infiltration, formation of proinflammatory, anti-inflammatory and profibrotic cytokines, fibrosis, and fatty infiltration in liver tissues with various liver disorders were also systematized. Data on the effect of SB on the state of the gut, gut microbiota, intestinal permeability, and small intestinal bacterial overgrowth in various liver disorders were also analyzed (Table 2 and Table 3).

EASs and RCTs were evaluated together.

### 3.1. Effect of Saccharomyces boulardii on the ALT/AST Levels in Liver Disorders

SB reduced the elevated ALT and AST levels due to acute mouse liver injury caused by D-galactosamine [22]. SB also reduced elevated ALT and AST levels in a rat carbon tetrachloride-induced cirrhosis model [27,30]. However, SB did not have a significant effect on the levels of ALT and AST in decompensated cirrhosis patients [16].

SB reduced the elevated serum ALT levels in streptozotocin-induced MASLD in mice without affecting the AST levels, which were not elevated in this model [20]. SB also reduced the elevated serum AST levels in rats with high-fat diet-induced MASLD without significantly affecting the elevated levels of ALT [26]. SB reduced the elevated ALT levels in a mouse model of methionine-choline-deficient diet-induced MASLD [29]; however, AST levels were not examined in this study [29].

There was no significant effect of SB on the blood ALT and AST levels in experimental obstructive jaundice in rats [18].

### 3.2. Effect of Saccharomyces boulardii on the GGT/ALP Levels in Liver Disorders

There was no significant effect of SB on the ALP levels in experimental obstructive jaundice in rats [18]. In one RCT, SB decreased the level of ALP and GGT in decompensated cirrhosis patients [16].

### 3.3. Effect of Saccharomyces boulardii on the Bilirubin Levels in Liver Disorders

SB had no significant effect on the blood bilirubin levels in experimental obstructive jaundice in rats [18] or in an RCT with decompensated cirrhosis patients [16].

In three RCTs, the addition of SB to phototherapy resulted in a more rapid and significant reduction in neonatal hyperbilirubinemia [21,25,31]. Another RCT did not establish any effect of SB in the treatment of this disease [28]. Unfortunately, these data could not be used for meta-analysis due to different data presentation formats.

### 3.4. Effect of Saccharomyces boulardii on the Serum Albumin Levels in Liver Disorders

SB increased the serum albumin levels that were reduced in cirrhosis in one RCT [16], but not in a rat cirrhosis model [30].

### 3.5. Effect of Saccharomyces boulardii on the Indicators of Lipid and Carbohydrate Metabolism in Liver Disorders

SB had no effects on fasted glycemia and insulinemia in the type 2 diabetes db/db mice model with MASLD [24]. However, it reduced hyperglycemia and protein glycation in the liver in streptozotocin-induced experimental diabetes and MASLD in mice [20].

SB did not have a significant effect on the serum triglyceride levels in one experimental model of MASLD [26], and on serum levels of glucose, cholesterol, and triglycerides in an RCT with decompensated cirrhosis patients [16].

### 3.6. Effect of Saccharomyces boulardii on the Manifestations of Cirrhosis and Hemodynamics

SB reduced the severity of many manifestations of cirrhosis (ascites, hepatic encephalopathy, hyperdynamic circulation, hypoalbuminemia, thrombocytopenia, hyponatremia) and lowered the Child–Pugh scores in one RCT with decompensated cirrhosis patients [16]. However, SB did not have a significant effect on the severity of esophageal varices in this RCT. In addition, the greatest effect of SB on reducing the Child–Pugh scores was observed in individuals with severe bacterial translocation, the biomarker of which was presepsin [32].

### 3.7. Effect of Saccharomyces boulardii on Systemic Inflammation in Liver Disorders

SB reduced the serum concentrations of tumor necrosis factor alpha (TNF-α), IL-1β, IL-6, and IL-4 in animals with MASLD [24,26].

SB reduced the serum TNF-α and IL-6 levels in experimental carbon tetrachloride-induced cirrhosis in rats [27,30] and also reduced the level of CRP in one RCT with decompensated cirrhosis patients [16].

### 3.8. Effect of Saccharomyces boulardii on Oxidative Stress in Liver Disorders

SB prevented the development of oxidative stress in liver tissues in response to experimental Giardia infection and metronidazole administration in gerbils [19]. SB also increased the activity of superoxide dismutase without significantly affecting the activity of catalase in the liver in experimental Giardia infection and healthy gerbils [19].

SB prevented decreases in the liver activity of superoxide dismutase and glutathione peroxidase in streptozotocin-induced MASLD in mice [20].

SB also prevented increases in the blood levels of the oxidative stress biomarker malondialdehyde in carbon tetrachloride-induced cirrhosis in rats [27].

### 3.9. Effect of Saccharomyces boulardii on Liver Inflammation and Injury

SB reduced venous blood congestion and hydropic degeneration in the liver in streptozotocin-induced MASLD in mice [20], and also reduced the liver amounts of biomarkers of macrophage infiltration (cluster of differentiation 11c (CD11c) and F4/80 mRNA levels), monocyte chemoattractant protein 1 (MCP-1), and IL-1β mRNA in mice with hereditary MASLD [24]. In addition, in one mouse model of MASLD, SB reduced hepatitis activity according to the NAS scale, production in the liver of TNF-α, IL-1β, interferon-γ, and IL-10 as well as the inflammatory cell chemoattractant CCL-2 and macrophage (Kupffer cells) biomarker F4/80 [29]. The histological severity of steatosis and inflammation decreased without changing the degree of ballooning in the latter case [29].

SB reduced the liver expression of the TNF-α and IL-6 genes in carbon tetrachloride-induced cirrhosis in rats [27]. In addition, SB reduced the severity of inflammation, hemorrhage, and necrosis in the liver as manifestations of acute mouse liver injury caused by D-galactosamine [22], and also reduced the liver infiltration of neutrophilic granulocytes, lymphocytes, and plasmocytes caused by Salmonella enteritidis infection in mice [23].

### 3.10. Effect of Saccharomyces boulardii on Liver Steatosis

SB reduced the body and liver weights, masses of total, subcutaneous, visceral, and liver fat, and histological liver steatosis scores in animals with experimental MASLD [24,26,29].

### 3.11. Effect of Saccharomyces boulardii on Liver Fibrosis

SB reduced the increased expression of genes of transforming growth factor β (profibrogenic cytokine), α-smooth muscle actin (a biomarker of connective tissue-forming activated hepatic stellate cells), and collagen in the liver as well as the severity of liver fibrosis in experimental cirrhosis in rats [27] and in a mouse model of MASLD [29].

### 3.12. Effect of Saccharomyces boulardii on the Gut Microbiota in Liver Disorders

Despite some inconsistencies in the results, most studies in the present review found that SB reduced the levels of harmful Escherichia (of the Proteobacteria phylum) in the gut microbiota (Table 4). These bacteria produce an active endotoxin, the penetration of which into the human body through an apparently intact intestinal wall (bacterial translocation) causes inflammation in the intestine, liver, and throughout the body [33,34,35]. In addition, most studies showed an increased abundance of Bacteroidetes in the gut microbiota as a result of SB usage. These bacteria have a variety of effects on the macroorganism including active participation in the formation of SCFAs [36], which strengthen the intestinal barrier [37].

SB restored the reduced gut microbiota alpha-diversity in a mouse model of MASLD [29].

### 3.13. Effect of Saccharomyces boulardii on Small Intestinal Bacterial Overgrowth in Liver Disorders

SB eliminated small intestinal bacterial overgrowth in the RCT with decompensated cirrhosis patients, and this was associated with improvements in liver function and better medium-term life prognoses [17].

### 3.14. Effect of Saccharomyces boulardii on Gut Structure and Permeability in Liver Disorders

In animal models of MASLD, SB was found to increase the cecum weight and reduce the severity of intestinal villi disorganization and atrophic changes as well as the formation of TNF-α and IL-1β in the walls of the small and large intestines [24,26,29].

SB prevented an increase in the permeability of the intestinal barrier and a decrease in the formation of tight junction protein ZO-1 in the proximal small intestine in a mouse model of MASLD [29]. SB did not have a significant effect on the reduced levels of occludin in the intestinal mucosa in experimental MASLD in rats [26]. However, SB did prevent increased intestinal permeability in experimental cirrhosis in rats [27].

SB contributed to a reduction in the elevated blood levels of I-FABP (a biomarker of intestinal epithelial damage) in experimental MASLD in rats [26].

### 3.15. Effect of Saccharomyces boulardii on Bacterial Translocation in Liver Disorders

SB reduced the severity of bacterial translocation to the liver, spleen, mesenteric lymph nodes, and blood in experimental obstructive jaundice in rats. Moreover, its protective effect was at the same level as the effect of antibiotics [18]. SB also abated the translocation of Salmonella enterica to the liver in mice [23].

Finally, SB reduced endotoxemia in the models of cirrhosis and MASLD [26,27,29,30] and in decompensated cirrhosis [17].

## 4. Discussion

The results of our systematic review indicate that SB has the ability to restore the gut–liver axis, which is disrupted in chronic liver diseases. This effect has been most effectively revealed in experimental studies of MASLD and in cirrhosis. In cirrhosis, the positive effect of SB on the gut–liver axis has been demonstrated in both experimental models and RCTs involving humans. However, the effect of SB on the gut–liver axis in alcoholic liver disease and many other chronic liver diseases has not yet been studied, and this represents a challenge for future research. The positive effect of SB on the gut–liver axis in MASLD should also be tested in future RCTs involving humans.

**Figure 2:**
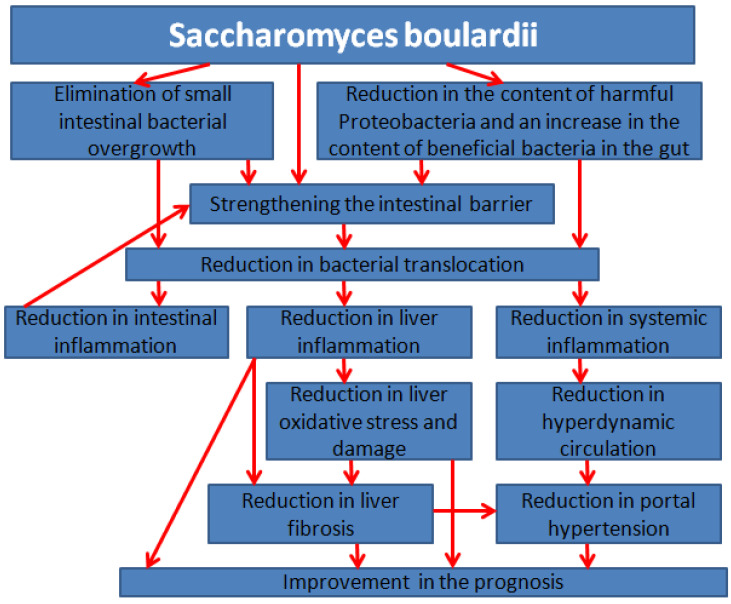
Proposed mechanism of restoration of the gut–liver axis by *Saccharomyces boulardii*.

Based on the data obtained in the present systematic review, the mechanism of the positive effect of SB in chronic liver diseases can be presented briefly as follows:

Due to its antagonistic and symbiotic properties, SB eliminates small intestinal bacterial overgrowth and modulates the composition of gut microbiota, reducing the content levels of harmful Proteobacteria and increasing the content levels of beneficial bacteria, which strengthen the intestinal barrier. Strengthening the intestinal barrier and reducing the content of harmful bacteria reduces the severity of bacterial translocation into the intestinal wall, liver, and systemic circulation, thereby reducing the severity of systemic, intestinal, and hepatic inflammation. Reducing the severity of intestinal inflammation, in turn, strengthens the intestinal barrier. In addition, reducing hepatic inflammation reduces the severity of oxidative stress, damage, and fibrosis in the liver. In decompensated cirrhosis, reducing systemic inflammation reduces the severity of hyperdynamic circulation; this then reduces the severity of portal hypertension, which reduces the severity of ascites, hepatic encephalopathy, hypoalbuminemia, and hypersplenism, improving the prognoses for patients (Figure 2).

Every year, the amount of information confirming the important role of the gut–liver axis in the development of various liver diseases is growing. Therefore, the use of drugs that can affect it is an important task of modern hepatology [3,4,5,6,7]. Such drugs include antibiotics, prebiotics, probiotics, postbiotics (metabiotics), and synbiotics [3,4,5,6,7]. Antibiotics kill intestinal bacteria with varying degrees of selectivity, allowing resistant strains to grow. Their widespread and long-term use in liver diseases is undesirable, since it leads to increased resistance to them and worse changes in the composition of the gut microbiota in most cases due to their non-selective effect, leading to the development of antibiotic-mediated gut dysbiosis [38]. Prebiotics are nutrients that are not absorbed by the human body but are used by intestinal bacteria as an energy source. Most of them have a non-specific effect and can be used not only by beneficial, but also by harmful bacteria [4]. Postbiotics are dead bacteria and their components that have a variety of effects on the body, but their use in liver diseases has been virtually unstudied [4]. Probiotics appear to be the most promising of these drugs as they have shown high safety including long-term use and good compliance [8]. These drugs are live bacteria that, when taken orally, have various beneficial effects on the human body. Probiotics have already shown their effectiveness in the treatment of hepatic encephalopathy in cirrhosis, while their effect on other manifestations of cirrhosis is still poorly understood [8]. Probiotics have shown a positive effect in experimental models of alcoholic liver disease and MASLD. Some of these effects have been confirmed in RCTs [8]. It should be emphasized that different probiotics have different effects and the effect obtained from the use of one probiotic should not be mechanistically extended to other probiotics. In this regard, it is relevant to create such systematic reviews that summarize data on the effect of not all probiotics, but only one selected probiotic microorganism. Moreover, different strains of one probiotic microorganism can also have different effects. In our case, the most studied strain of SB, namely the strain CNCM I-745, was used in most publications. Thus, most of the conclusions of this systematic review apply specifically to this strain, and should be transferred to other strains with caution.

The strength of our review is that it is the first to summarize all of the available information on the effect of SB on liver disorders. The limitations of this review are the small number of RCTs included and the small number of diseases in both RCTs and EASs. New RCTs on the use of SB in cirrhosis are needed to confirm the obtained results, and new RCTs on the use of SB in MASLD are required to confirm the results obtained in EASs. In addition, EASs and RCTs on the use of SB in the complex treatment of chronic hepatitis B, autoimmune, and cholestatic liver diseases are also required.

## 5. Conclusions

SB shows promise as a complementary treatment for chronic liver diseases. Its effectiveness in the treatment of cirrhosis has been demonstrated in EASs and RCTs. However, its efficacy in the treatment of MASLD has only shown been in EASs. Further RCTs are needed to verify its effects in MASLD and other chronic liver diseases.

## Figures and Tables

**Table 1:** Characteristics of the experimental animal studies included in the systematic review.

Study	Organisms	CNCM I-745	Disease	Count of Participants	Groups
Geyik 2006 [18]	Rats	+	ObstrJ	12 × 5	N/C/C + SB/C + AB/C + AB + SB
Ribeiro 2020 [19]	Gerbils	+	Giardiasis	8 × 8	N/SB/AB/SB + AB/C/ C + SB/C + AB/C + AB + SB
Barssotti 2021 [20]	Mice		MASLD	(6–9) × 4	N/SB/C/C + SB
Yu 2017 [22]	Mice	+	DILI	5 × 3	N/C/C + SB
Wu 2014 [23]	Mice		Sal	No data	N/C/C + SB/C + OP
Everard 2014 [24]	Mice	+	MASLD	15 × 2	C/SB
Liu 2016 [26]	Rats		MASLD	12 × 3	N/C/SB
Li 2015 [27]	Rats	+	Cirrhosis	8 + 10 + 8+8	N/C/SB/SB *
Yang 2022 [29]	Mice	+	MASLD	5/5/9/10	N/N + SB/C/SB
Pang 2015 [30]	Rats		Cirrhosis	10 × 3	N/C/SB

AB—antibiotic; C—control (disease without tested interventions); DILI—D-galactosamine-induced liver injury; MASLD—metabolic dysfunction–associated steatotic liver disease; N—normal (without disease); ObstrJ—obstructive jaundice; OP—other probiotics; Sal—Salmonella infection; SB—*Saccharomyces boulardi*. *—another regime of using the SB.

**Table 2:** Parameters analyzed in the included experimental animal studies.

Study	ALT/ AST	GGT/ ALP	Bilirubin	Serum Albumin	Serum Glucose	Serum Lipids	Systemic Inflammation	Oxidative stress	Liver Inflammation	Liver Steatosis	Liver Fibrosis	Gut Micro-biota	Gut Structure and Permeability	Bacterial Translocation
Geyik 2006 [18]	+	+	+											+
Ribeiro 2020 [19]								+						
Barssotti 2021 [20]	+				+			+	+					
Yu 2017 [22]	+								+			+		
Wu 2014 [23]									+					+
Everard 2014 [24]					+		+		+	+		+	+	
Liu 2016 [26]	+					+	+			+		+	+	+
Li 2015 [27]	+						+	+	+		+	+	+	+
Yang 2022 [29]	+								+	+	+	+	+	+
Pang 2015 [30]	+			+			+							+

**Table 3:** Characteristics of the randomized controlled trials included in the systematic review.

Study	CNCM I-745	Disease	Count of Participants	Groups	ALT/ AST	GGT/ ALP	Bilirubin	Serum Albumin	Serum Glucose/Lipids	Cirrhosis Manifes-tations	Hemodynamics	Prognosis	Systemic Inflammation	Gut Micro-Biota	SIBO	Bacterial Translocation
Tang 2020 [21]	+	NHB	63 + 61	SB/C			+							+		
Suganthi 2016 [25]		NHB	86 + 95	SB/C			+									
Serce 2015 [28]	+	NHB	98 + 81	SB/C			+									
Hu 2022 [31]	+	NHB	55 + 45	SB/C			+									
Maslennikov 2022 [16]	+	Cirrhosis	24 + 16	SB/Pl	+	+	+	+	+	+	+		+			
Efremova 2024 [17]	+	Cirrhosis	20 + 13	SB/Pl								+			+	+

C—control (disease without tested interventions); NHB—neonate hyperbilirubinemia; Pl—placebo; SB—*Saccharomyces boulardi*; SIBO—small intestinal bacterial overgrowth.

**Table 4:** Changes in the composition of the gut microbiota in liver disorders as a result of *Saccharomyces boulardii* (SB) intake.

Disorders	Taxa Whose Abundances Were Increased by SB in the Gut Microbiota	Taxa Whose Abundances Were Decreased by SB in the Gut Microbiota
Human neonate hyperbilirubinemia [21]	Bacteroidetes	Firmicutes, Proteobacteria, Escherichia coli, and Staphylococcus
Acute mouse liver injury caused by D-galactosamine [22]	Bacteroidetes, Bacteroidaceae, and Clostridiaceae	Firmicutes, Proteobacteria, Alcaligenaceae, Anaeroplasmataceae, Caulobacteraceae, and Rikenellaceae
Mouse hereditary diabetes and MASLD [24]	Bacteroidetes and Bacteroidaceae	Firmicutes, Proteobacteria, Tenericutes, Porphyromonadaceae, Anaeroplasma, Anaerotruncus, Dorea, Odoribacter, Oscillospira, Parabacteroides, Prevotella, and Ruminococcus
High-fat diet-induced MASLD in rats [26]	Bacteroides	Escherichia coli
Mouse model of methionine-choline-deficient diet-induced MASLD [29]	Bacteroides, Lachnospiraceae, Atopobiaceae, Ruminococcaceae, Faecalibaculum, and Blautia	
Carbon tetrachloride-induced cirrhosis in rats [27]	Clostridium leptum	Escherichia coli and Enterococcus faecalis
